# Protective Effect of a Prime-Boost Strategy with the Ts87 Vaccine against *Trichinella spiralis* Infection in Mice

**DOI:** 10.1155/2014/326860

**Published:** 2014-08-28

**Authors:** Yuan Gu, Bin Zhan, Yaping Yang, Xiaodi Yang, Xi Zhao, Lei Wang, Jing Yang, Kuo Bi, Yunyun Wang, Xinping Zhu

**Affiliations:** ^1^Department of Parasitology, School of Basic Medical Sciences, Capital Medical University, Beijing 100069, China; ^2^Department of Pediatrics, Section of Tropical Medicine, Baylor College of Medicine, Houston, TX 77030, USA

## Abstract

Trichinellosis is a widespread zoonosis primarily caused by *Trichinella spiralis*. Mucosal immunity is crucial for preventing *Trichinella spiralis* infection. In our previous study, a DNA vaccine with the *Trichinella* antigen Ts87 delivered by an attenuated *Salmonella typhimurium* elicited partial protection against *Trichinella spiralis* infection in mice. In the current study, to elicit a more robust immune response and develop a potent vaccination strategy against trichinellosis, a heterologous prime-boost vaccination regimen for Ts87 was used in mice and the protective efficacy was evaluated compared to the homologous DNA prime-boost or protein prime-boost immunization alone. The results revealed that the DNA-prime/protein-boost vaccination with Ts87 induced higher levels of both humoral and cellular immune responses. The challenge results showed that mice with the DNA-prime/protein-boost vaccination displayed higher muscle larval reduction than those immunized with DNA prime-boost or protein prime-boost. The results demonstrated that mice vaccinated with Ts87 in a DNA-prime/protein-boost strategy effectively elicited a local IgA response and mixed Th1/Th2 immune response that might be responsible for improved protection against *Trichinella spiralis* infection.

## 1. Introduction

Trichinellosis is a major food-borne zoonosis and human infection has been reported in 55 countries around the world [1]. Human trichinellosis is characterized by high fever, facial edema, and myositis, which may be serious, particularly in elderly patients [2]. The nematode* Trichinella spiralis* is the most common cause of human trichinellosis [3]. Outbreaks of trichinellosis have been regularly reported during the past two centuries and this parasitic disease is emerging or reemerging in some areas of the world [4–6]. Trichinellosis is not only a public health hazard but also an economic problem for livestock production and food safety [7]. Consequently, there is an urgent need for vaccines to control the infection.

The occurrence of trichinellosis in humans is strictly related to cultural food practices, including the consumption of raw or undercooked meat containing encapsulated* Trichinella* parasite larvae [7]. The infective muscle larvae are released from the muscle tissue in the stomach and migrate to the small intestine where the larvae develop into adult worms. The adult females produce newborn larvae, which penetrate the intestine and migrate to muscle tissue where they form cysts. Therefore, the intestinal mucosa is likely to be the first barrier in protecting the host against* Trichinella* infection. In our previous studies, an immunodominant antigen, Ts87, was cloned from* T. spiralis* [8], and vaccination with the recombinant Ts87 protein (rTs87) produced partial protection in immunized mice [9, 10]. To induce an IgA response in the intestinal mucosa, the Ts87 DNA was transformed into attenuated* S. typhimurium*. Mice vaccinated orally with the attenuated* Salmonella*-delivered Ts87 DNA vaccine exhibited a strong local IgA response and partial protection against* T. spiralis* infection [11].

Although the mucosal immunity induced by the attenuated* Salmonella*-delivered DNA vaccine produced partial protection against* Trichinella* infection, the systemic immune response to the Ts87 DNA vaccination was not high enough and the elicited protection was limited [11]. An effective vaccine usually requires a more optimal immunization regimen in the form of a prime-boost. A heterologous prime-boost regimen can be more immunogenic than a homologous prime-boost regimen [12]. In recent years, many promising results and significant protection have been reported for viral, bacterial, and parasitic infections using the heterologous prime-boost regimen [13–15]. In this study, to elicit a more robust immune response, including local mucosal IgA production and a more potent vaccination strategy against Trichinellosis, a heterologous prime-boost vaccination regimen with Ts87 DNA and rTs87 was used and the protective immunity induced by this regimen was evaluated.

## 2. Materials and Methods

### 2.1. Parasites

*T. spiralis* (ISS 533) parasites were originally isolated from a swine source in the Heilongjiang province of China and maintained by serial passage in female ICR mice. Each mouse was orally infected with 400* T. spiralis* larvae. The muscle larvae (ML) were recovered from infected mice using a modified pepsin-hydrochloric acid digestion method as described by Gamble et al. [16, 17].

### 2.2. Mice/Ethics Statement

Female, 6-7 week-old BALB/c mice were purchased from the Laboratory Animal Services Center of Capital Medical University (Beijing, China). All experimental procedures were reviewed and approved by the Capital Medical University Animal Care and Use Committee and were consistent with the NIH Guidelines for the Care and Use of Laboratory Animals.

### 2.3. Ts87 DNA Vaccine

DNA encoding the full-length Ts87 was cloned into the eukaryotic expression vector pVAX1, and the recombinant pVAX1-Ts87 plasmid DNA was transformed into an attenuated* S. typhimurium* SL7207 strain as a DNA vaccine (SL7207/pVAX1-Ts87) as described previously [11].

### 2.4. Recombinant Ts87 Protein (rTs87)

The rTs87 was expressed in* E. coli* BL21 (DE3) with a His-tag at the C-terminus and was purified using Ni-affinity chromatography (Novagen, USA) as described previously [11].

### 2.5. Immunization Regimens

In this study, BALB/c mice were vaccinated with either rTs87 or Ts87 DNA transformed attenuated* S. typhimurium* with different prime-boost strategies. For the DNA prime-protein boost regimen, a group of 12 mice were immunized orally with 1 × 10^8^ cells of SL7207/pVAX1-Ts87 as described previously [11] and then boosted twice at 2-week intervals with 100 *μ*g rTs87 emulsified with the water-in-oil adjuvant ISA 50 V2 (SEPPIC, France) intramuscularly [18]. All prime-boost regimens are described in Table 1. Two weeks after the last boost, six mice from each group were sacrificed. The serum, intestinal lavage fluid, spleen, and mesenteric lymph nodes (MLNs) were collected to evaluate the humoral and cellular immune responses. Mice immunized three times with PBS were used as a blank control.

### 2.6. Antibody Responses

The levels of antigen-specific total IgG and subtype IgG1 and IgG2a antibodies in the sera of the immunized mice were determined using a modified indirect enzyme-linked immunosorbent assay (ELISA) as described previously [19]. Briefly, 96-well microtiter plates (Costar) were coated with rTs87 (10 *μ*g/mL) and blocked with 5% fetal bovine serum (FBS) in PBS. For total IgG detection, the plates were incubated with sera at different dilution and then incubated with HRP-conjugated goat anti-mouse IgG. For the isotype-specific ELISA, after incubation with the mouse sera samples (1 : 200 dilution), the plates were incubated with goat anti-mouse IgG1 or IgG2a (BD Pharmingen, USA). Then, HRP-conjugated rabbit anti-goat IgG antibodies (BD Biosciences, USA) were added. The ELISA plates were developed with o-phenylenediamine dihydrochloride substrate (OPD, Sigma, USA) and read at 492 nm.

### 2.7. Measurement of Total IgA in Intestinal Washes

The intestinal lavage fluid was prepared as described previously [11]. Briefly, for each sacrificed mouse, 10 cm of the small intestine beginning at the gastroduodenal junction was cut, and the interior of the small intestine was flushed twice with a total of 2 mL of cold PBS. After centrifugation at 800 ×g for 10 min, the supernatants were harvested and stored at −80°C until use. The total intestinal IgA was assessed with a sandwich-type ELISA by trapping the intestinal mucosal IgA as described previously [20].

### 2.8. T Cell Proliferation

A T cell proliferation assay was performed using the CellTiter 96 Aq_ueous_ One Solution Cell Proliferation Assay (Promega, USA). Briefly, 5 × 10^5^ splenocytes in 100 *μ*L of RPMI-1640 were* in vitro* stimulated with 100 *μ*L of rTs87 (10 *μ*g/mL) for 72 h. Then, 40 *μ*L of the CellTiter 96 AQ_ueous_ One Solution Reagent was added to each well and incubated for 1–4 hours at 37°C. The stimulation index (SI) was calculated as the ratio of the mean absorbance of the stimulated/unstimulated wells.

### 2.9. Cytokine Assays

An enzyme-linked immunospot assay (ELISPOT) was used to detect IFN-*γ*, IL-4, IL-6, and IL-10 secreted by the lymphocytes isolated from the spleen and MLNs of immunized mice according to the manufacturer's instructions (BD Biosciences, USA) [21]. Briefly, the mice were sacrificed two weeks after the last boost and the lymphocytes from the spleen and the MLNs were aseptically isolated. The wells of MultiScreen-IP Filter Plates for ELISPOT (Millipore, USA) were coated with the capture antibody (anti-mouse IFN-*γ*, IL-4, IL-6, and IL-10; BD Biosciences, USA) at a 1 : 200 dilution in PBS and incubated overnight at 4°C. The plates were washed once with RPMI 1640 medium (Gibco, USA) with 10% FBS and blocked with the same medium for 2 h at room temperature. A total of 1 × 10^6^ lymphocytes for IL-4, IL-6, and IL-10 or 5 × 10^5^ lymphocytes for IFN-*γ* were added to each well. The rTs87 was added to the well at a final concentration of 10 *μ*g/mL and stimulated for 48 h. Concanavalin A (ConA, Sigma, USA; 5 *μ*g/mL) was used as a nonspecific positive control. The detection antibody (biotinylated anti-IFN-*γ*, IL-4, IL-6, and IL-10 antibody; BD Biosciences Pharmingen, USA) was added at 1 : 200 in 100 *μ*L of dilution buffer (PBS containing 10% FBS) and incubation was continued for 2 h. After incubation with 100 *μ*L of streptavidin-HRP for 1 h (BD Biosciences, USA), the plates were developed with 100 *μ*L of a 3-amino-9-ethylcarbazole substrate solution (20 *μ*L of an AEC chromogen for each 1 mL of substrate, BD ELISPOT AEC substrate set; BD Biosciences, USA) for 1–5 min. The spots corresponding to the number of IFN-*γ*, IL-4, IL-6, and IL-10-secreting cells were counted automatically with a CTL ELISPOT reader and analyzed using the ImmunoSpot image analyzer software v4.0.

### 2.10. Evaluation of Larval Burden

Two weeks after the final boost, the remaining 6 mice from each group were challenged with 400* T. spiralis* muscle larvae. Six weeks after the challenge, the mice were sacrificed. The larvae in the muscle from each mouse were collected and counted as described previously [17]. Reductions in the larval burden were calculated as follows: worm burden reduction rate (%) = (1 − mean number of larvae per gram muscle in vaccinated mice/mean number of larvae per gram muscle in control mice) × 100%.

### 2.11. Statistical Analysis

All of the data were evaluated by one-way ANOVA using the SPSS 17.0 software. The data are expressed as the means ± standard error (SE). *P* < 0.05 was considered statistically significant.

## 3. Results

### 3.1. Serological Immune Response

**Figure 1:**
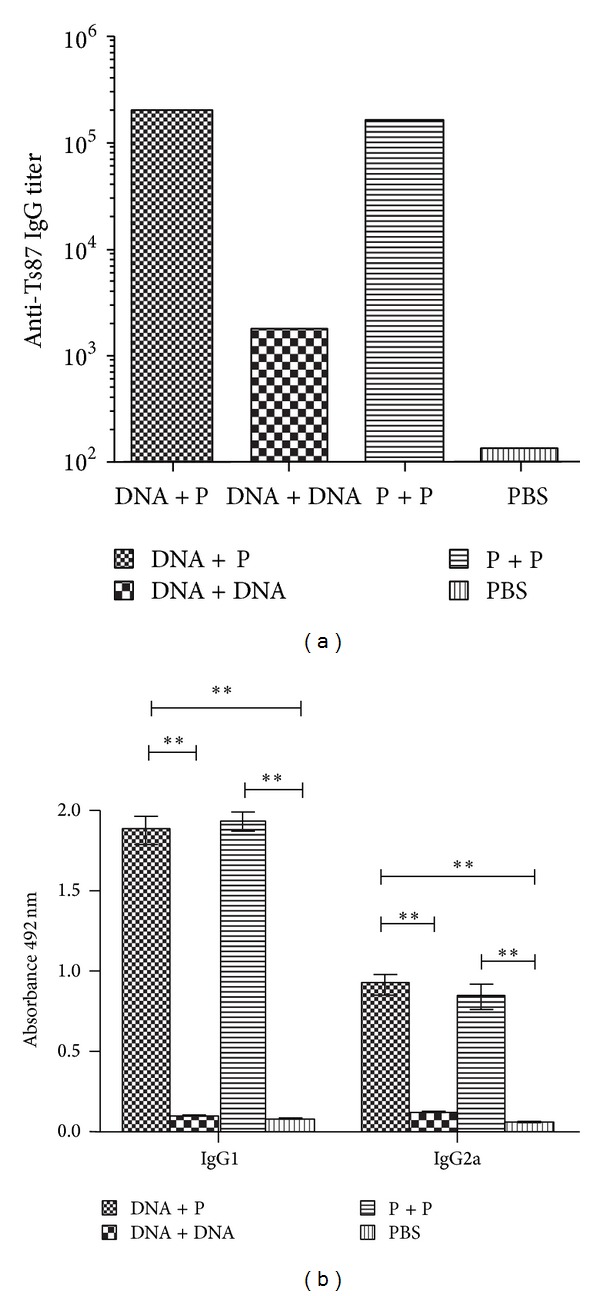
rTs87-specific total IgG and subtype IgG1 and IgG2a levels detected in the sera of immunized mice. The mouse sera were collected two weeks after the 3rd immunization and were measured by ELISA. The rTs87-specific total IgG is shown as the geometric mean titers within the group (Figure 1(a)). **P < 0.01. The subtype IgG1 and IgG2a levels are shown as the mean absorbance values ± SE.

Mice immunized orally with Ts87 DNA-attenuated* S. typhimurium* (DNA) and then boosted intramuscularly twice with rTs87 (P) produced much higher levels of total IgG, IgG1, and IgG2a compared to the group boosted with Ts87 DNA alone (*P* < 0.01, Figure 1). The group immunized with rTs87 and boosted with the same protein twice also produced high levels of total IgG, IgG1, and IgG2a. The production of IgG1 and IgG2a indicates a mixed Th1(IgG2a) or Th2-like (IgG1) responses, with Th2 predominant.

### 3.2. Mucosal IgA Response

**Figure 2:**
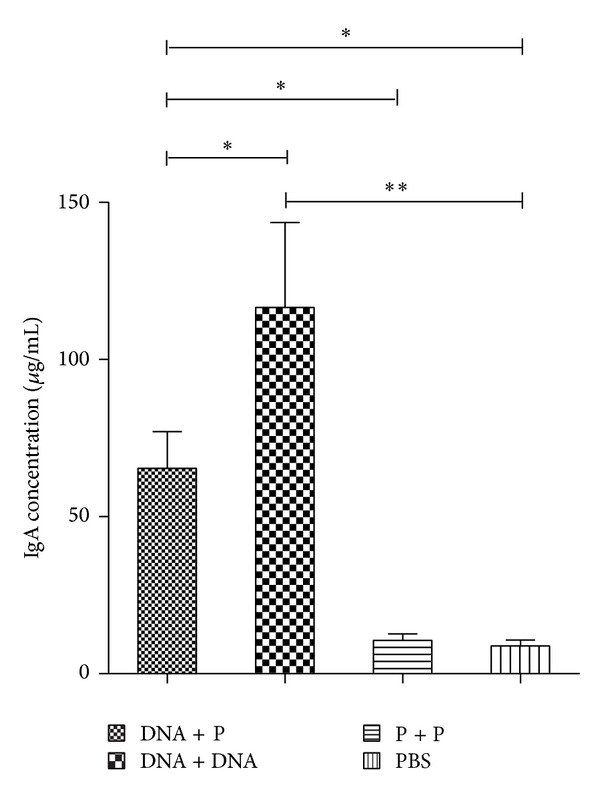
The total secretory IgA in the intestinal washes of vaccinated mice measured by sandwich ELISA. The IgA level was increased in the mice immunized with the DNA-prime/protein-boost (DNA + P) compared to those immunized with rTs87 (P + P) or PBS (*P* < 0.05). The IgA level was significantly higher in mice with the DNA prime-boost (DNA + DNA) compared with the DNA-prime/protein-boost (DNA + P) (*P* < 0.05). **P < 0.01. *P < 0.05. The results are presented as the mean ± SE for 6 mice per group.

The total intestinal mucosa IgA was measured by sandwich ELISA. The secretory IgA level was significantly increased in the mucosa of mice immunized orally with the Ts87 DNA vaccine, either boosted with the same DNA (DNA + DNA) or with the recombinant protein (DNA + P) compared to the group with the protein prime-boost (P + P). However, the highest level of secretory IgA was observed in the DNA immunized group boosted with the same DNA carried with* Salmonella* bacteria compared to the protein boosted group (*P* < 0.05) (Figure 2). There was no significant secretion of mucosal IgA in protein immunized group compared to the PBS control group.

### 3.3. Cytokine Profiles

**Figure 3:**
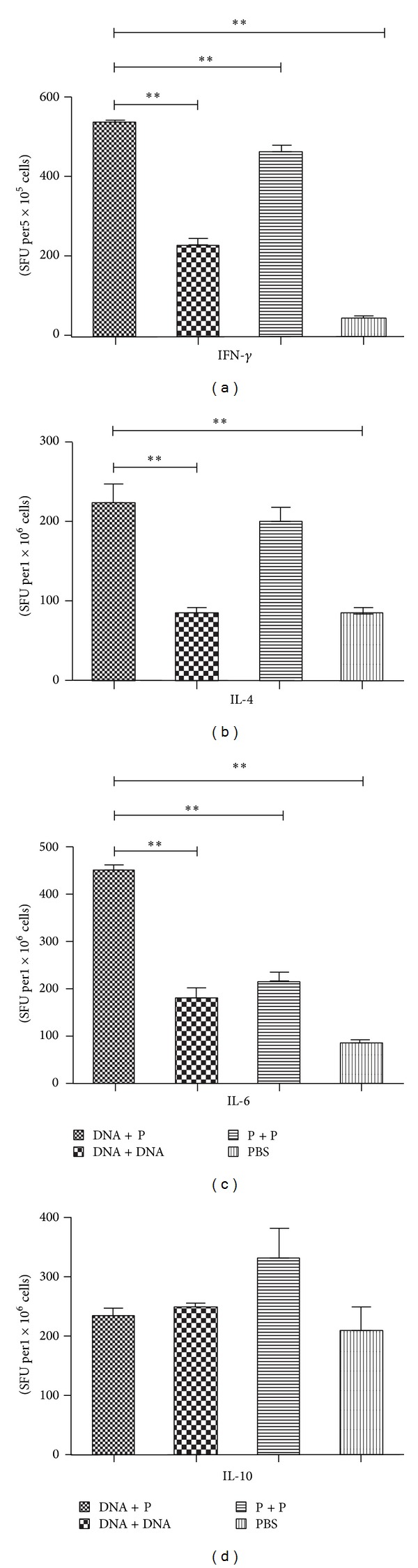
IFN-*γ*, IL-4, IL-6, and IL-10 secreted by splenocytes isolated from mice immunized with Ts87 in different prime-boost regimens were detected by ELISPOT. **P < 0.01. The results are presented as the mean ± SE for 6 mice per group.

**Figure 4:**
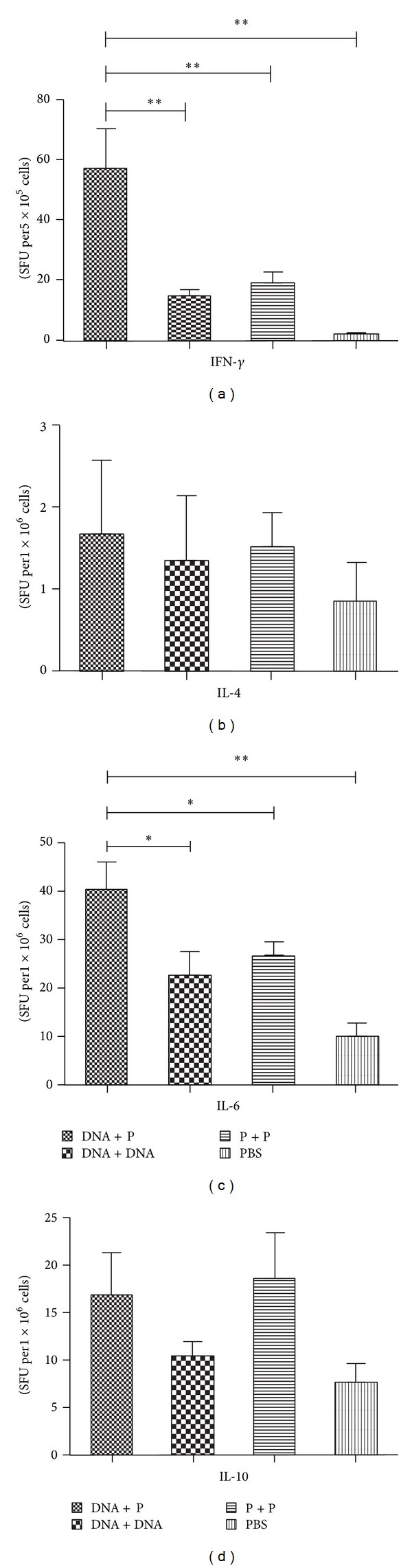
IFN-*γ*, IL-4, IL-6, and IL-10 secreted by lymphocytes of MLNs isolated from mice immunized with Ts87 in different prime-boost regimens were detected by ELISPOT. The IL-4 levels in the MLN cells of all four groups were low (less than five spots, Figure 4(b)). **P < 0.01. ^∗^ *P* < 0.05. The results are presented as the mean ± SE for 6 mice per group.

The ELISPOT assay was used to detect the cytokines IFN-*γ*, IL-4, IL-6, and IL-10 secreted by the lymphocytes isolated from the spleen and MLNs two weeks after the 3rd immunization. The IFN-*γ* and IL-6 levels were significantly increased in the lymphocytes isolated from both the spleen and MLNs of mice immunized with the Ts87 DNA-prime/protein-boost compared to homologous prime-boost immunization regimens (Figures 3(a), 3(c), 4(a), and 4(c)). The IL-4 level was significantly increased in the groups immunized with the DNA-prime/protein-boost or the protein prime-boost in splenocytes compared to the groups immunized with the DNA prime-boost or PBS control (Figure 3(b)). However, the secretion of IL-4 was hardly tested in the MLN cells (less than five spots, Figure 4(b)). Although a higher level of IL-10 was consistently observed in the group immunized with the rTs87 protein prime-boost compared to the other immunization groups, this change was not statistically significant (Figures 3(d) and 4(d)). No spots were detected in the unstimulated lymphocytes, whereas the positive spots were all high without exception in the ConA stimulated control groups (up to 400/5 × 10^5^ cells, data not shown).

### 3.4. Proliferative Responses of T Cells

**Figure 5:**
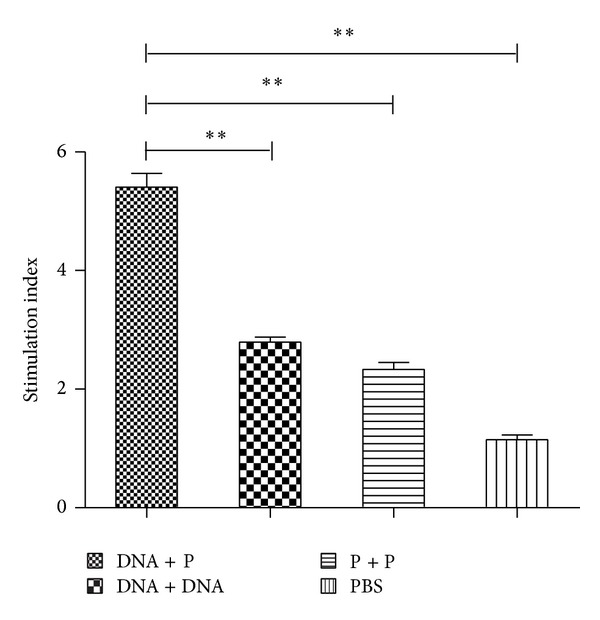
Proliferative responses of splenocytes upon stimulation of rTs87* in vitro*. The rTs87-stimulated T cell proliferation of splenocytes isolated from the DNA-prime/protein-boost (DNA + P) mice was significantly higher than that in other homologous prime-boost groups. **P < 0.01. The results are presented as the mean ± SE for 6 mice per group.

The rTs87-stimulated T cell proliferation of the splenocytes isolated from the DNA-prime/protein-boost mice was significantly higher than the other three groups (*P* < 0.01), indicating that heterologous immunization with a DNA vaccine-prime and recombinant protein-boost greatly enhanced the antigen-specific T cell proliferative response against rTs87 (Figure 5).

### 3.5. Protective Immunity

**Figure 6:**
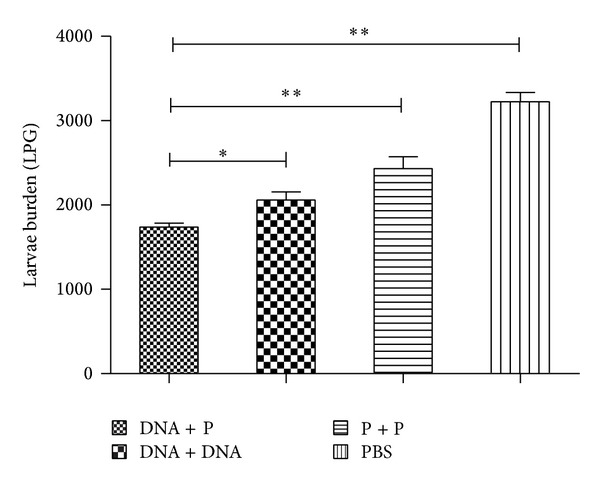
Protection elicited by immunization with different vaccination regimens. The larvae per gram muscle (LPG) were counted in the muscles of mice 6 weeks after a challenge with 400* T. spiralis* larvae. The mice immunized with the DNA-prime/protein-boost (DNA + P) displayed a 46.1% reduction in the muscle larval burden compared to the groups immunized with the homologous DNA prime-boost (36.2%, *P* < 0.05) and homologous protein prime-boost (24.6%, *P* < 0.01). **P < 0.01. *P < 0.05. The results are presented as the mean ± SE for 6 mice per group.

In comparison to the PBS control group, mice immunized with DNA prime-protein boost, DNA prime-boost, and protein prime-boost experienced 46.1%, 36.2%, and 24.6% reduction in muscle larval burden, respectively (Figure 6). There is a significant difference between the heterologous prime-boost vaccination regimen and the homologous DNA prime-boost (*P* < 0.05) or between the heterologous prime-boost vaccination regimen and homologous protein prime-boost immunization (*P* < 0.01). These results indicate that the DNAprime/protein-boost vaccination induced significantly better protective immunity than the homologous DNA prime-boost or protein prime-boost regimens against* T. spiralis* infection in BALB/c mice.

## 4. Discussion

DNA vaccination becomes more attractive because of its ability to induce a broad range of immune responses and long-lasting immunity. However, DNA vaccines remain poorly immunogenic compared to protein vaccines [22]. An effective vaccine usually requires more than one immunization in the form of a prime-boost. Traditionally, the same vaccines are administered multiple times as homologous boosts. New findings suggest that the prime-boost can be performed with different types of vaccines containing the same antigens. This type of heterologous prime-boost can be more immunogenic than the homologous prime-boost and may elicit unique immune responses allowing for improved immunogenicity and/or protection against viral, bacterial, and parasitic infections [21, 23–25].

The DNA plus protein vaccination strategy utilizes the benefits of DNA and protein vaccines to effectively induce both cell-mediated immunity and antibody responses against invading organisms [26]. Human studies have also shown superior immune responses during mixed modality prime-boost [27, 28]. The objective of the present study was to explore the protective efficacy and characteristics of the immune response elicited by a DNA prime followed by a protein boost compared to a homologous DNA or protein immunization alone. The kinetics of the mucosal and systemic antibody secretion, patterns of antibody subtype production, cytokine production by the spleen, MLN lymphocyte, and protective effect of this DNA-prime/protein-boost regimen against* T. spiralis* infection were evaluated in mice in this study.

Mucosal immune responses act as the first barrier of defense against* T. spiralis*. The mucosal IgA response, when adequately induced, can impede the establishment of infective* Trichinella* parasites in the mouse intestine [29]. Intranasal immunization with a 30-mer peptide of a 43 kDa* Trichinella* antigen induced protective immunity against* T. spiralis* infection accompanied by the secretion of mucosal IgA [30]. Intraperitoneal injection of an IgA monoclonal antibody against the* Trichinella* parasite also protected mice from infection with infective larva [29]. The DNA vaccine delivered by attenuated* S. typhimurium* produced long-lasting mucosal IgA and systemic immune responses and provides an efficient vaccination platform, particularly for intestinal infections in which local immunity is essential for protection [31–33]. In our previous study, oral vaccination with Ts87 DNA delivered by* S. typhimurium* induced significant intestinal IgA secretion and considerable protective immunity against the challenge of* T. spiralis* infective larva [11]. Compared to the homologous DNA prime-boost vaccination, the heterologous Ts87 DNA-prime and protein-boost regimen examined in this study produced significant high level of systemic antibody responses, including increased total IgG and subtypes IgG1 and IgG2a and significantly greater protection against* T. spiralis* larval challenge compared to homologous DNA or protein prime-boost regimens. The greater protection in the mice immunized with the Ts87 DNA-prime and protein-boost regimen (46.1%) compared to the mice immunized with the homologous DNA prime-boost (36.2%) is also associated with more robust cellular responses demonstrated by greater lymphocyte proliferation upon specific antigen stimulation and the higher level of INF-*γ* secreted by both splenocytes and MLNs. It has been demonstrated that the combined Th1 and Th2 immune responses are important for immunity against* T. spiralis* infection [19, 34, 35], even though it is believed that the Th2 response is essential for protective immunity to gastrointestinal (GI) helminth infections [36]. In this study, mice immunized with the DNA-prime/protein-boost produced not only a stronger Th2-associated immune response (IgG1 antibody, secretory mucosal IgA, IL-4, and IL-6), but also a Th1-like response evidenced by high titers of IgG2a antibody and IFN-*γ*. The results indicate that this heterologous immunization regimen of a DNA-prime/protein-boost with a Ts87 vaccine produced a mixed Th1 and Th2 immune response that may contribute to greater protection than homologous DNA or protein prime-boost alone.

High levels of IgA secretion in the mucosal tissue were also observed in the mice vaccinated with the oral Ts87 DNA-prime and intramuscular protein-boost, although the IgA level was not as high as those orally vaccinated three times with Ts87 DNA. IL-4, IL-6, and IL-10 are associated with murine IgA responses [37]. IL-6 has been identified to be the most effective terminal differentiation factor for IgA-committed B cells to become IgA-producing cells in both human and mouse systems [38]. In this study, we also observed high levels of IL-6 secreted by splenocytes and MLNs from mice vaccinated with the Ts87 DNA-prime/protein-boost than other groups with homologous prime-boost vaccination regimens. Significantly higher levels of IL-4 were secreted by the splenocytes in the mice vaccinated with the DNA-prime/protein-boost and protein prime-boost than those vaccinated with the DNA prime-boost or PBS control. Although it is believed that IL-10 plays an essential role in IgA B-cell differentiation in humans [39], in the present study, there was no significant difference in the level of IL-10 secreted by lymphocytes from the mice vaccinated with the DNA-prime/protein-boost regimen and the other immunization regimens. High levels of IL-6 correlated with the elevated intestinal mucosal IgA level upon DNA-prime/protein-boost immunization, indicating that IL-6 may contribute more to the intestinal mucosa IgA response.

In conclusion, the objective of this study was to improve the efficacy of the Ts87 vaccine using a heterologous prime-boost vaccination strategy. The results revealed that the DNA-prime/protein-boost vaccination regimen for Ts87 induced both humoral and cellular immune responses against* T. spiralis* infection, which was associated with high levels of mucosal secreted IgA, serological IgG (total IgG, IgG1, and IgG2a), and lymphocyte secreted IFN-*γ*, IL-4, and IL-6. Challenge experiments further demonstrated that the DNA-prime/protein-boost vaccination with Ts87 produced significantly greater muscle larval reduction than the traditional homologous prime-boost vaccination. Therefore, the Ts87 vaccine using DNA-prime/protein-boost vaccination produced more effective vaccine efficacy against trichinellosis. Additional studies are needed including optimizing the inoculation dosage, route, immunization sequence, and timing of delivery for this prime-boost vaccination strategy.

## Figures and Tables

**Table 1:** Immunization regimen.

Group (prime_boost)	Prime	1st boost	2nd boost
DNA + P	SL7207/pVAX1-Ts87 (orally)	rTs87 (intramuscularly)	rTs87 (intramuscularly)
DNA + DNA	SL7207/pVAX1-Ts87 (orally)	SL7207/pVAX1-Ts87 (orally)	SL7207/pVAX1-Ts87 (orally)
P + P	rTs87 (intramuscularly)	rTs87 (intramuscularly)	rTs87 (intramuscularly)
PBS	PBS	PBS	PBS
